# Semantic-Guided Transformer Network for Crop Classification in Hyperspectral Images

**DOI:** 10.3390/jimaging11020037

**Published:** 2025-01-26

**Authors:** Weiqiang Pi, Tao Zhang, Rongyang Wang, Guowei Ma, Yong Wang, Jianmin Du

**Affiliations:** 1 College of Intelligent Manufacturing and Elevator, Huzhou Vocational and Technical College, Huzhou 313099, China; piweiqiang20171@163.com (W.P.); rongyang1987@126.com (R.W.); magw666@163.com (G.M.); 13185285607@163.com (Y.W.); 2 College of Mechanical and Electrical Engineering, Inner Mongolia Agricultural University, Hohhot 010018, China; nndjwc202@imau.edu.cn

**Keywords:** hyperspectral image classification, transformer, deep learning, attention mechanism, convolutional neural network

## Abstract

The hyperspectral remote sensing images of agricultural crops contain rich spectral information, which can provide important details about crop growth status, diseases, and pests. However, existing crop classification methods face several key limitations when processing hyperspectral remote sensing images, primarily in the following aspects. First, the complex background in the images. Various elements in the background may have similar spectral characteristics to the crops, and this spectral similarity makes the classification model susceptible to background interference, thus reducing classification accuracy. Second, the differences in crop scales increase the difficulty of feature extraction. In different image regions, the scale of crops can vary significantly, and traditional classification methods often struggle to effectively capture this information. Additionally, due to the limitations of spectral information, especially under multi-scale variation backgrounds, the extraction of crop information becomes even more challenging, leading to instability in the classification results. To address these issues, a semantic-guided transformer network (SGTN) is proposed, which aims to effectively overcome the limitations of these deep learning methods and improve crop classification accuracy and robustness. First, a multi-scale spatial–spectral information extraction (MSIE) module is designed that effectively handle the variations of crops at different scales in the image, thereby extracting richer and more accurate features, and reducing the impact of scale changes. Second, a semantic-guided attention (SGA) module is proposed, which enhances the model’s sensitivity to crop semantic information, further reducing background interference and improving the accuracy of crop area recognition. By combining the MSIE and SGA modules, the SGTN can focus on the semantic features of crops at multiple scales, thus generating more accurate classification results. Finally, a two-stage feature extraction structure is employed to further optimize the extraction of crop semantic features and enhance classification accuracy. The results show that on the Indian Pines, Pavia University, and Salinas benchmark datasets, the overall accuracies of the proposed model are 98.24%, 98.34%, and 97.89%, respectively. Compared with other methods, the model achieves better classification accuracy and generalization performance. In the future, the SGTN is expected to be applied to more agricultural remote sensing tasks, such as crop disease detection and yield prediction, providing more reliable technical support for precision agriculture and agricultural monitoring.

## 1. Introduction

Crop classification is an important application of hyperspectral remote sensing technology [1]. Thanks to the numerous narrow spectral bands, hyperspectral imaging has been widely applied in various fields such as environmental monitoring, agriculture, mineral exploration, and urban planning [2,3,4,5]. Hyperspectral images capture continuous spectral information of target objects across a wide spectral range, enabling the detection of subtle spectral reflectance differences on material surfaces [6,7,8]. However, due to the high morphological and color similarity of crops, as well as the complexity of the background (e.g., the mixture of weeds and crops), fine-grained crop classification becomes a significant challenge.

In recent years, with the advancement of computational power and the development of machine learning algorithms [9,10], particularly deep learning methods, the rapid progress of crop hyperspectral image classification technology has been facilitated [11,12,13]. These technological advancements have not only improved classification accuracy but also made significant strides in handling high-dimensional data and reducing computational complexity [14,15]. Deep learning-based methods can automatically extract deep abstract features of ground objects from hyperspectral images, which has led to widespread applications in the remote sensing field [16,17,18]. Among these, convolutional neural networks (CNNs) can effectively capture local details and deep semantic features in images through convolution operations [19]. Zhang et al. [20] used 2D CNNs to effectively extract complex features of desert grasslands. Falaschetti et al. [21] utilized CNNs for pest and disease recognition in plant leaf images. Although CNNs have achieved remarkable results in extracting image features, they face certain limitations in handling long-range dependencies and capturing global features, which restricts their application in hyperspectral image classification [22]. This is especially true in crop hyperspectral images, where the high color similarity between crops and weeds makes it difficult to accurately distinguish between different crop types.

Recently, image classification methods based on the transformer model have garnered widespread attention from researchers [23]. The transformer model, through its self-attention mechanism, can directly model global dependencies and capture richer feature representations, making it particularly suitable for handling the spatial complexity and high-dimensional spectral characteristics of hyperspectral data [24]. Unlike CNNs, which rely on convolution operations, transformers aggregate features from different scales and spatial locations through self-attention and multi-head attention mechanisms, demonstrating stronger expressiveness and flexibility in hyperspectral image classification [25]. This novel approach has provided new ideas and tools for hyperspectral image classification, driving further advancements in the field. Yang et al. [26] demonstrated superior classification performance by applying the transformer model to hyperspectral images compared to CNNs. Additionally, some studies have shown that combining CNNs’ local feature extraction capability with the transformer’s ability to capture long-range dependencies can further improve hyperspectral image classification performance. For example, Zhang et al. [27] introduced convolution operations within the multi-head self-attention mechanism to better capture local–global spectral features of hyperspectral images. Wang et al. [28] improved the ResNet-34 network to extract features from hyperspectral images and then passed these features into a transformer to capture global dependencies, thus enhancing classification performance. Although the combination of CNNs and transformers effectively improves hyperspectral image classification performance, these methods overlook the issue of spatial information loss due to the serialization processing of transformer models.

To address the learning deficiencies of transformer models in spatial information, researchers have proposed several effective solutions that combine CNNs and transformers to improve model performance. For example, Zhao et al. [29] achieved feature fusion across different layers by interactively combining CNNs and transformers. This approach effectively combines CNNs’ advantages in local feature extraction with transformers’ capabilities in global feature modeling, thereby enhancing the learning of spatial information. However, this fusion method still faces challenges such as increased model complexity and reduced training efficiency. Li et al. [30] proposed a hierarchical feature fusion strategy based on 3D-CNNs, aimed at joint learning of spatial–spectral features. This method captures spatial and spectral information in hyperspectral images more comprehensively through 3D convolution operations. However, in practical applications, the fixed-size image sampling strategy still introduces a large amount of background heterogeneous information, which may interfere with the model’s ability to predict crop labels. Additionally, due to the spectral similarity between the background and crops, this background interference becomes especially pronounced in various scenarios, leading to a decline in classification performance.

To better address these challenges, this paper proposes a semantic-guided transformer network (SGTN) for the fine-grained classification of crop hyperspectral images. SGTN introduces a multi-scale spatial–spectral information extraction (MSIE) module, specifically designed to effectively model crop variations at different scales, thereby reducing the impact of background information. This module not only captures the changing characteristics of crops at multiple scales but also enhances the richness and accuracy of features, laying a solid foundation for subsequent classification tasks. Furthermore, SGTN includes a semantic-guided attention (SGA) module, which improves the model’s sensitivity to crop semantic information. By precisely focusing on crop regions, this module effectively reduces background interference and improves classification accuracy. Through the integrated use of the MSIE and SGA modules, SGTN excels in feature extraction and fusion, significantly addressing the limitations encountered by existing methods in hyperspectral image classification tasks. This paper aims to enhance the fine-grained classification accuracy of crops, providing technical support for the development of precision agriculture. The main contributions of this article are as follows:

(1) The SGTN model is proposed for hyperspectral image crop classification, achieving a balance between accuracy and speed.
(2) The MSIE and SGA modules are designed, where MSIE is aimed at extracting multi-scale information of crops, while SGA enhances the semantic representation of the crops.
(3) Extensive experiments are conducted on three benchmark datasets, and the proposed model achieves state-of-the-art performance compared to the most advanced methods.

The remaining structure of this paper is organized as follows: Section 2 details the proposed SGTN model. Section 3 presents the experimental results on the three datasets. Section 4 concludes the paper.

## 2. Methodology

**Figure 1:**
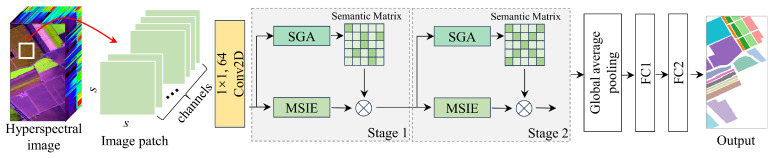
The SGTN model framework, where the symbol ⊗ represents element-wise multiplication.

The SGTN structure proposed in this study is shown in Figure 1. The network consists of two stages of feature extraction layers. Each stage includes the MSIE and SGA modules, where the MSIE module is primarily used to extract spatial information of crops at different scales, and the SGA module guides the model to learn more semantically relevant features. After feature extraction, a global average pooling function is applied to aggregate the spatial information. This is followed by two fully connected layers that output the crop categories. It is important to note that 64 convolution filters of size 1 × 1 are used to reduce the spectral dimensionality. In the fully connected (FC) layers, the number of neurons in the layers is distributed as 32 and the corresponding number of crop categories. Overall, the feature extraction layer of the model can be divided into two branches. The main branch is the MSIE module, which is primarily responsible for learning crop features, while the other branch is the SGA module, which focuses on semantic guidance to improve the feature learning of the main branch. Notably, the model’s input patches are sampled using a sliding window approach with a fixed spatial size, aimed at better capturing spatial information. In general, the label of the central pixel of the patch is typically considered the classification label of the entire patch, representing the category of the most representative pixel within the patch. Specifically, if the spatial size of a patch is s×s, the label of the central pixel is usually assigned as the label for the entire patch, ensuring an effective representation of the region’s features and its category.

### 2.1. Multi-Scale Spatial–Spectral Information Extraction

**Figure 2:**
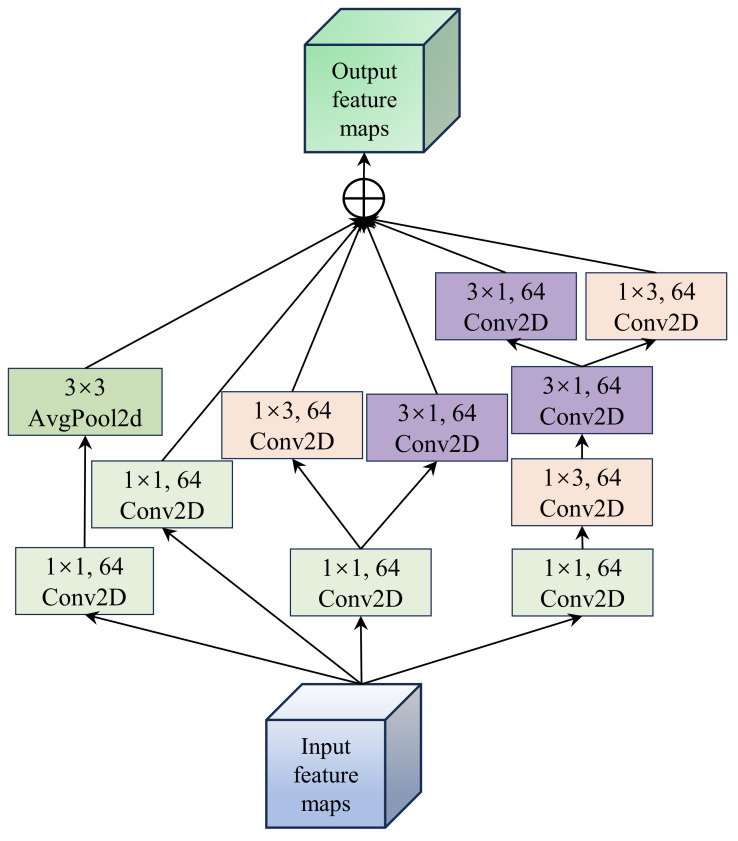
Illustration of the MSIE module, where the symbol ⊕ denotes element-wise summation.

The MSIE module, as the primary feature extraction structure of the model, effectively captures the spatial information of crops, as shown in Figure 2. This module extracts features through parallel multiple branches and then aggregates and outputs the corresponding feature maps from each branch. In the first branch, a 1 × 1 convolution kernel is first used to extract spectral details from the image, followed by a 3 × 3 2D average pooling function to aggregate the spatial information. To ensure spatial scale invariance, zero padding is applied to the 2D pooling function. For the second branch, a 1 × 1 convolution kernel is employed to better capture the nonlinear spectral variation details. The third branch follows a similar structure to the first branch, where a 1 × 1 convolution kernel is used for spectral feature learning, followed by 1 × 3 and 3 × 1 convolution kernels to capture edge detail features while reducing model parameters. In contrast to the third branch, the fourth branch considers the impact of series and parallel connections on model learning. Therefore, it uses concatenated 1 × 3 and 3 × 1 convolution kernels to further enhance the model’s learning capability. Specifically, a 1 × 1 convolution kernel is first applied, followed by concatenated 1 × 3 and 3 × 1 convolution kernels, and then parallel feature extraction from both convolution kernels. It is important to note that, to ensure the feature maps from different branches can be correctly aggregated, the number of filters in all convolution kernels is set to 64.

Overall, the MSIE module is designed to achieve efficient feature representation through multi-scale feature extraction and computational optimization. This module utilizes multiple parallel branches to extract features at different scales, combining 1 × 1 convolutions to capture local details and 3 × 3 convolutions to extract local spatial information, thereby realizing the fusion of multi-scale features. Notably, the use of parallel branches allows for the processing of different types of features, adapting to the diverse patterns and scale variations within the image. Additionally, the traditional 3 × 3 convolution is decomposed into two unidirectional convolutions (i.e., 1 × 3 and 3 × 1 convolutions) to achieve the same effect while reducing the number of parameters to improve efficiency. Furthermore, performing convolution operations in both horizontal and vertical directions enhances the boundary feature information of the crops.

### 2.2. Semantically Guided Attention Module

**Figure 3:**
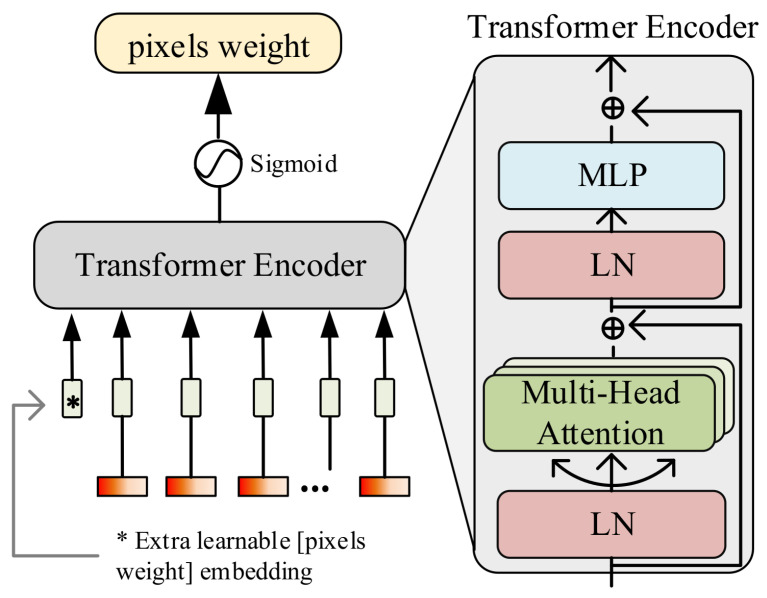
Illustration of the SGA module.

The SGA module is designed by improving the transformer model, incorporating multi-head self-attention, multilayer perceptron (MLP), and layer normalization (LN), as shown in Figure 3. This module aims to guide the model in strengthening the semantic output for crops. In the SGA module, the original positional encoding function in the transformer is discarded, and a learnable pixel weight parameter $w_{p}\in R^{s^{2}\times 1}$ is embedded along the channel dimension, where *s* denotes the spatial size. The mathematical representation is as follows:

(1) $$x_{p}=\mathrm{Concat}\left(x,w_{p}\right)$$

where Concat(·) represents the concatenation of data along the specified dimension. *x* denotes the original input data to the model, and $x_{p}$ represents the concatenated result, with dimensions $s^{2}\times \left(c+1\right)$, where *c* is the number of channels in the data. Additionally, considering the feature redundancy caused by a large number of channels, this paper uses a learnable parameter $w_{c}\in R^{1\times c}$ to filter important spectral bands. The mathematical expression is as follows:

(2) $$x_{c}=\mathrm{Sigmoid}\left(w_{p}\right)\times x$$

where Sigmoid(·) represents the Sigmoid activation function.

In the transformer model, before the input is passed into the multi-head self-attention mechanism, it undergoes LN to reduce covariate shift during training and accelerate the convergence of training. In the self-attention layer, three different linear transformations are used to generate three new vector representations: the query vector (*Q*), the key vector (*K*), and the value vector (*V*). For each query vector, its dot product with all key vectors is calculated to measure the similarity between the query and each key. The attention weights are then computed based on the results of these dot products, and these weights are used in matrix multiplication with the value vectors to obtain the final output. The calculation formula for the attention weights is as follows:

(3) $$\mathrm{Attention}\left(Q,K,V\right)=\mathrm{softmax}\left(\frac{QK^{T}}{\sqrt{d_{k}}}\right)V$$

where $d_{k}$ represents the dimensionality of the key vector, while *Q*, *K*, and *V* are the query matrix, key matrix, and value matrix, respectively. The softmax function is applied to the scaled dot product of *Q* and *K* to produce a set of attention weights, which are then used to compute the weighted sum of the value vectors. This process allows the model to focus on different parts of the input sequence when making predictions.

In multi-head self-attention, the attention mechanism is executed independently multiple times, with each attention head using different linear transformation parameters to generate distinct queries, keys, and values. These independent attention heads allow the model to focus on different parts or features of the input sequence simultaneously, capturing various relationships and patterns from multiple perspectives. The formula for multi-head self-attention is as follows:

(4) $$\mathrm{MultiHead}\left(Q,K,V\right)=\mathrm{Concat}\left({\mathrm{head}}_{1},\ldots ,{\mathrm{head}}_{h}\right)W_{O}$$

where ${\mathrm{head}}_{i}=Attention\left(Q_{i},K_{i},V_{i}\right)$, where h is the number of heads, and $W_{O}$ is the linear transformation matrix used to transform the output of multiple heads back to the original dimensions after splicing. The function Concat(·) represents concatenating the results of each self-attention computation along the feature dimension *c* to obtain the final output of the multi-head self-attention mechanism.

After obtaining the output from the self-attention layer, the model’s nonlinear expressiveness is enhanced through the MLP. It is worth noting that a residual connection is used in the transformer encoder structure to facilitate feature reuse within the network, allowing the model to learn more efficiently. After the transformer encoding, the pixel weight parameter $w_{p}$ is extracted and reshaped to a spatial size of s×s, resulting in weights with the same spatial dimensions as the input image. Notably, this weight is normalized using a Sigmoid activation function to obtain the weight size for each spatial pixel. Based on this weight $w_{p}$, the network is continuously guided during the learning process to focus on the true semantic categories of the image, thereby minimizing background interference. Finally, this weight is element-wise multiplied with the main branch to enhance the model’s semantic output, which in turn improves crop recognition accuracy in complex background scenarios.

## 3. Results and Analysis

All experiments in this study were conducted on hardware consisting of an Intel(R) Xeon(R) CPU E5-2682 V4, an NVIDIA GeForce RTX 3060-12G GPU, and 30 GB of memory. The programming language used was Python 3.8, and the deep learning framework was PyTorch 1.12.1. To effectively evaluate the classification performance of the model, three evaluation metrics were selected: overall accuracy (OA), average accuracy (AA), and Kappa coefficient.

In the experiment, cross-entropy was selected as the loss function, the batch size was set to 64, Adam was used as the optimizer, the learning rate was 0.001, and the number of iterations was 100. The SGA module in the model was set to a depth of one layer, and the number of attention heads in the self-attention mechanism was set to one. To eliminate experimental variability, the results were averaged over five repeated experiments.

### 3.1. Datasets

**Figure 4:**
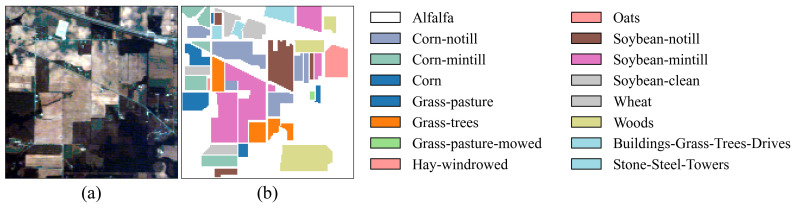
False-color image and ground truth for the IP dataset. (**a**) False-color image. (**b**) Ground truth.

**Figure 5:**
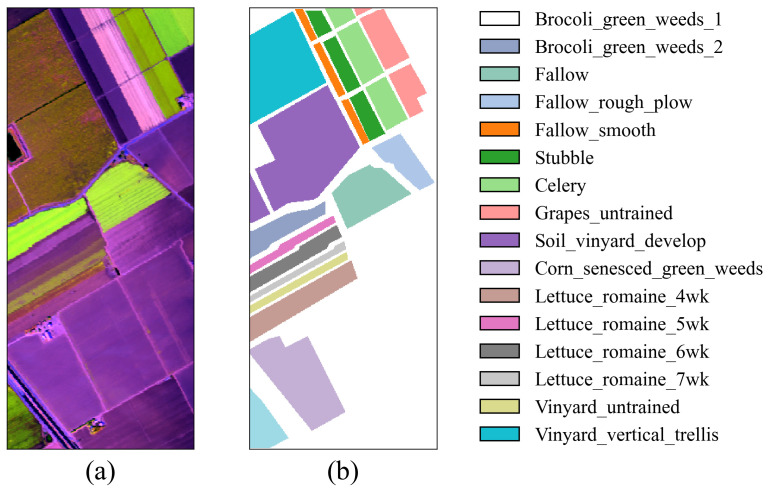
False-color image and ground truth for the SA dataset. (**a**) False-color image. (**b**) Ground truth.

**Figure 6:**
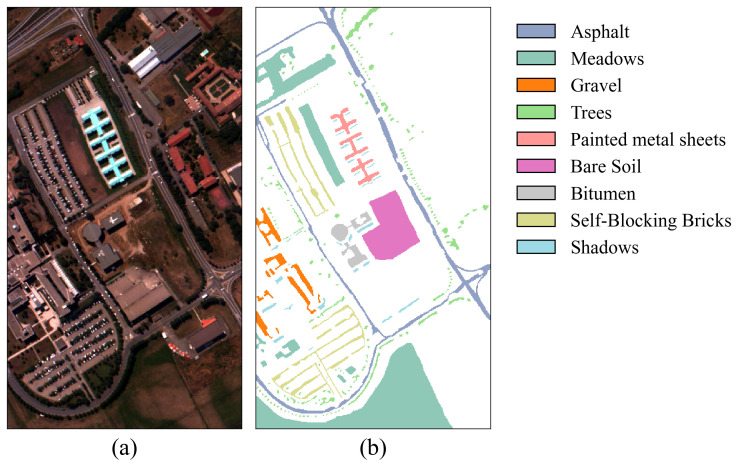
False-color image and ground truth for the PU dataset. (**a**) False-color image. (**b**) Ground truth.

In the experiments, two crop hyperspectral image benchmark datasets were used: Indian Pines (IP) and Salinas (SA) [31]. Additionally, the Pavia University (PU) dataset was chosen to assess the model’s generalization capability. The IP dataset is a typical crop dataset collected from the Indian Pines test site in Northwest Indiana, USA, using the AVIRIS sensor (Jet Propulsion Laboratory, Pasadena, CA, USA). It has a spatial size of 145 × 145 and includes 220 spectral bands. After removing bands that cannot be reflected by water, 200 bands were retained for analysis. This dataset contains 16 crop categories and a total of 10,249 labeled samples. The SA dataset, also obtained by the AVIRIS sensor, was collected near Salinas, CA, USA. The image has a spatial size of 512 × 217, with an original 224 spectral bands. After removing noise-affected bands, 204 valid bands were retained for analysis. This dataset includes 16 crop categories and a total of 54,129 labeled samples. The PU dataset was obtained by the ROSIS sensor and collected near the University of Pavia, Italy. It has a spatial size of 610 × 340 and contains 103 spectral bands. This dataset includes nine categories and a total of 42,776 labeled samples. In the experiments, for the IP dataset, 10%, 10%, and 80% of the samples were randomly selected for the training set, validation set, and test set, respectively. For the PU and SA datasets, 1%, 1%, and 98% of the samples were randomly selected for the training set, validation set, and test set, respectively. The sample divisions for the three datasets are shown in Table 1, Table 2 and Table 3, and the false color images and ground truth are shown in Figure 4, Figure 5 and Figure 6.

### 3.2. Impact of Space Size

**Figure 7:**
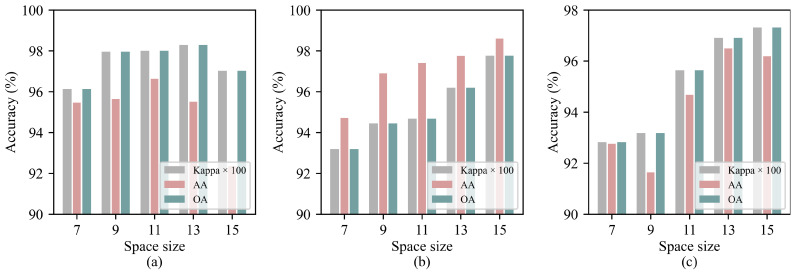
Experimental results for different spatial sizes on the three datasets: (**a**) IP. (**b**) SA. (**c**) PU.

Different spatial sizes have a significant impact on the model’s classification performance. Generally, as the spatial size increases, the spatial information contained in the image becomes more abundant. However, this also introduces more redundant information, which may negatively affect the model’s classification performance. Therefore, to evaluate the impact of different spatial sizes on the proposed SGTN model, five spatial scales in the range of 7, 9, 11, 13, 15 were selected for experimentation. The results are shown in Figure 7. From the figure, it can be observed that for all three datasets, classification accuracy tends to increase as the spatial size grows. For the IP dataset, the impact of different spatial sizes on the model accuracy is relatively small, with the classification accuracy reaching its maximum when the spatial size is 13. The PU and SA datasets are more sensitive to different input spatial sizes, with smaller spatial sizes resulting in lower classification accuracy. When the spatial size reaches 15, the classification accuracy of both datasets reaches its peak.

### 3.3. Ablation Experiment

To evaluate the impact of the SGA module on crop classification performance, ablation experiments were conducted, as shown in Table 4. From the table, it is evident that the classification accuracy has improved on all three datasets after the introduction of the SGA module. This demonstrates that the module can enhance the semantic relationships of crops in the image, thereby improving the model’s ability to capture the semantic features of each crop. On the IP dataset, the AA accuracy increased by 4.04% after introducing the SGA module, indicating a significant improvement, further validating the effectiveness of the SGA module. In addition, to verify the effectiveness of the MSIE module, it was replaced with a two-layer standard convolution for feature extraction. According to the experimental results, when the MSIE module was replaced with standard convolution, the accuracy significantly decreased on all three datasets, further demonstrating the effectiveness of the MSIE module. Overall, the SGTN model improves classification performance with only a slight increase in training time, effectively balancing the classification accuracy across all categories.

### 3.4. Classification Results

To validate the effectiveness of the proposed algorithm, six state-of-the-art hyperspectral image classification models were selected for comparison: DFFN [32], HSST [33], SSAN [34], SSFTTnet [24], MASSFormer [35], and MSSTT [36].

- DFFN (Deep Feature Fusion Network): This model optimizes convolutional layers, multilayer feature fusion, and PCA dimensionality reduction through residual learning, effectively enhancing classification accuracy, particularly excelling in small sample scenarios.
- HSST (Hierarchical Spatial–Spectral Transformer): An end-to-end hierarchical spatial–spectral transformer model that effectively extracts spatial–spectral features from hyperspectral data using multi-head self-attention (MHSA), while employing a hierarchical architecture to reduce the number of parameters.
- SSAN (Spectral–Spatial Attention Network): This model combines spectral and spatial modules and introduces an attention mechanism to suppress the influence of noisy pixels, effectively extracting discriminative spectral-spatial features from hyperspectral data.
- SSFTTnet (Spectral–Spatial Feature Tokenization Transformer): This model combines spectral-spatial feature extraction modules with a transformer architecture, utilizing a Gaussian-weighted feature tokenization module to convert shallow features into semantic features.
- MASSFormer (Memory-Augmented Spectral–Spatial Transformer): This model introduces a memory tokenizer (MT) and a memory-augmented transformer encoder (MATE) module to convert spectral–spatial features into memory tokens that store prior knowledge while extending multi-head self-attention (MHSA) operations to achieve more comprehensive information fusion.
- MSSTT (Multi-Scale Super-Token Transformer): This model includes a multi-scale convolution (MSConv) branch and a multi-scale super-token attention (MSSTA) branch to achieve both local and global feature extraction.

All models used the same structural parameters as those in the original papers, and experiments were conducted in the same experimental environment.

#### 3.4.1. Classification Results for the IP Dataset

**Figure 8:**
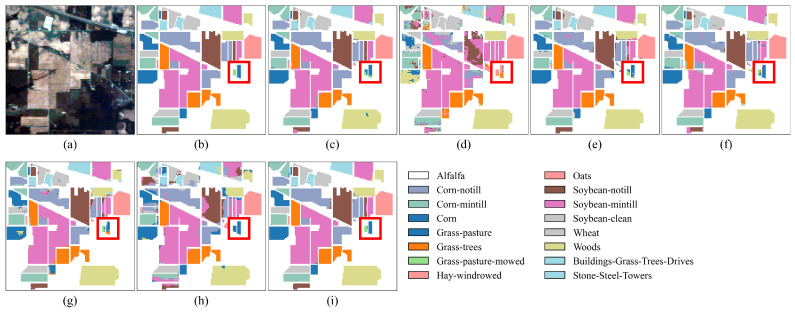
Classification result maps of different models on the IP dataset. (**a**) False-color image. (**b**) Ground truth. (**c**) DFFN. (**d**) HSST. (**e**) SSAN. (**f**) SSFTTnet. (**g**) MASSFormer. (**h**) MSSTT. (**i**) SGTN.

As shown in Table 5, the proposed SGTN achieves the highest classification accuracy among all comparison models, with OA, AA, and Kappa coefficients of 98.24%, 94.64%, and 0.9799, respectively. Specifically, compared to the model with the lowest accuracy, HSST, SGTN improves OA by 25.58%, showing a significant enhancement. Compared to the model with the highest classification accuracy, DFFN, SGTN improves OA, AA, and Kappa coefficients by 1.02%, 2.27%, and 1.16%, respectively, further demonstrating its excellent performance in crop classification. Additionally, from the perspective of AA, SGTN shows an improvement of up to 41.86%, with the smallest increase being 2.27%. This indicates that SGTN can effectively balance the classification accuracy across different crop categories. The primary reason for this performance is the ability of the SGA module to effectively extract semantic information, thereby enhancing the model’s semantic representation. Except for the crops “Alfalfa” and “Grass-pasture-mowed”, SGTN achieves over 90% classification accuracy for all other crop types. Furthermore, as observed in Figure 8, the classification results of SGTN are relatively smooth, and the model is capable of accurately identifying the spatial distribution of crops.

#### 3.4.2. Classification Results for the SA Dataset

**Figure 9:**
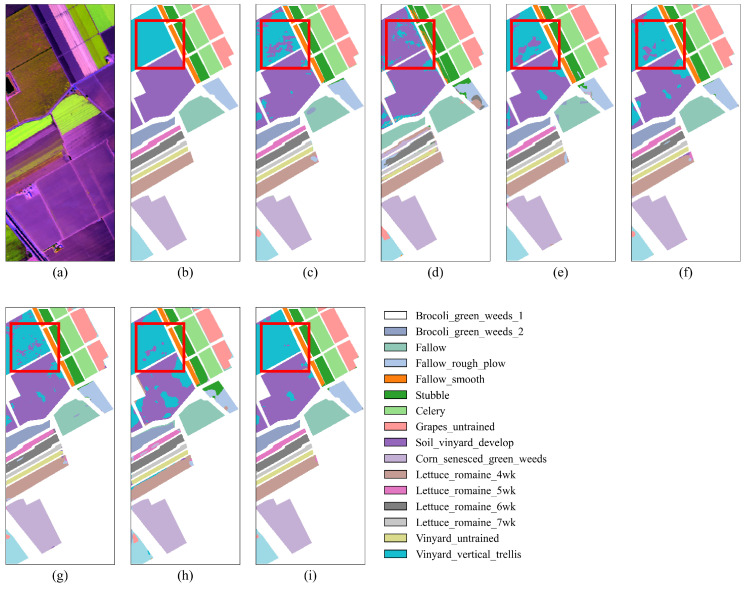
Classification result maps of different models on the SA dataset. (**a**) False-color image. (**b**) Ground truth. (**c**) DFFN. (**d**) HSST. (**e**) SSAN. (**f**) SSFTTnet. (**g**) MASSFormer. (**h**) MSSTT. (**i**) SGTN.

As shown in Figure 9, “Grapes_untrained” and “Vinyard_untrained” exhibit significant salt-and-pepper noise, particularly in the classification results of DFFN and HSST, as seen in Figure 9d,h. This misclassification is primarily due to the similarity in spectral and visual color information between these two grape-related categories, which makes them prone to confusion. In contrast, SGTN exhibits fewer misclassifications, attributed to the SGA module’s semantic guidance, which enhances the model’s semantic outputs. From Table 6, under the condition of only 1% training samples, SGTN achieved the highest classification performance, with OA, AA, and Kappa scores reaching 97.89%, 98.44%, and 0.9765, respectively. Compared to the other models, SGTN achieved improvements in OA, AA, and Kappa by 2.24–22.82%, 1.73–28.64%, and 2.49–25.80%, respectively. Observing the experimental results on the SA dataset, it can be seen that among the comparative models, the HSST model performs the worst, while the SSRN model shows better results. However, the overall classification performance of SSRN still falls short of SGTN.

#### 3.4.3. Classification Results for the PU Dataset

**Figure 10:**
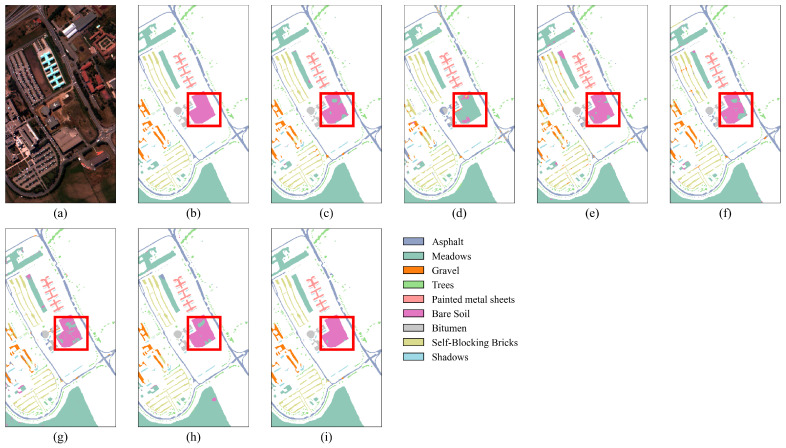
Classification result maps of different models on the PU dataset. (**a**) False-color image. (**b**) Ground truth. (**c**) DFFN. (**d**) HSST. (**e**) SSAN. (**f**) SSFTTnet. (**g**) MASSFormer. (**h**) MSSTT. (**i**) SGTN.

The results in Table 7 show that, with only 1% of the training samples, the proposed SGTN achieves the best classification accuracy, with OA, AA, and Kappa coefficients of 98.34%, 96.82%, and 0.9780, respectively. Additionally, the classification accuracy for all categories, except “Trees” and “Shadows”, exceeds 95%. Furthermore, as observed in Figure 10, for the recognition of “Bare Soil”, SGTN achieves better classification results with fewer misclassifications. Specifically, in Figure 10d, the HSST method performs poorly in recognizing “Bare Soil”. Compared to the other models, the SGTN model shows significant improvements in OA, AA, and Kappa, with accuracy gains ranging from 1.99% to 16.26%, 1.99% to 21.43%, and 2.64% to 22.23%, respectively. Overall, the classification results of the proposed SGTN are closer to the true ground truth of the dataset. These results further demonstrate the advanced generalization ability of SGTN.

### 3.5. Runtime Analysis

To assess the computational efficiency of the model, this section presents an analysis of the parameters and prediction time on three datasets, as shown in Table 8. The results indicate that SSAN has significantly more parameters than other models, while SSFTTnet has the fewest parameters. In terms of prediction time, except for MSSTT and HSST, the prediction time differences across the three datasets for the other methods are not significant, especially for the IP dataset. In contrast, the proposed SGTN model shows moderate performance in both parameters and prediction time, falling within the medium range. The model utilizes a self-attention mechanism to capture the semantic relationships in the image, which increases computational complexity but also leads to a significant improvement in classification accuracy. Overall, SGTN not only achieves the best classification results but also maintains a reasonable level of computational complexity, demonstrating balanced overall performance.

## 4. Conclusions

This paper proposes the SGTN model to enhance crop recognition accuracy. The model consists of two feature extraction layers and a classification layer. The feature extraction layers are composed of the MSIE and SGA modules, where the MSIE module extracts multi-scale information about crops, while the SGA module focuses on the semantic information of crops. To validate the effectiveness of the proposed algorithm, extensive experiments were conducted on the IP and SA crop benchmark datasets, with PU used to evaluate the model’s generalization ability. The results demonstrate that, compared to the state-of-the-art classification models, the proposed SGTN model achieves higher classification accuracy and generalization ability, showing the model’s significant potential in crop classification. Although this study achieved good results in crop classification, the quadratic complexity of the self-attention mechanism increased the model inference time. Therefore, future research will focus on improving the structure of the transformer model and utilizing various learning strategies to further enhance crop classification performance while reducing model complexity.

## Figures and Tables

**Table 1:** Sample division results for the IP dataset.

No.	Class	Train	Validation	Test	Total
1	Alfalfa	4	4	38	46
2	Corn-notill	142	128	1158	1428
3	Corn-mintill	83	74	673	830
4	Corn	23	21	193	237
5	Grass-pasture	48	43	392	483
6	Grass-trees	73	65	592	730
7	Grass-pasture-mowed	2	2	24	28
8	Hay-windrowed	47	43	388	478
9	Oats	2	1	17	20
10	Soybean-notill	97	87	788	972
11	Soybean-mintill	245	221	1989	2455
12	Soybean-clean	59	53	481	593
13	Wheat	20	18	167	205
14	Woods	126	113	1026	1265
15	Buildings-Grass-Trees-Drives	38	34	314	386
16	Stone-Steel-Towers	9	8	76	93
Total		1018	915	8316	10,249

**Table 2:** Sample division results for the SA dataset.

No.	Class	Train	Validation	Test	Total
1	Brocoli_green_weeds_1	20	19	1970	2009
2	Brocoli_green_weeds_2	37	36	3653	3726
3	Fallow	19	19	1938	1976
4	Fallow_rough_plow	13	13	1368	1394
5	Fallow_smooth	26	26	2626	2678
6	Stubble	39	39	3881	3959
7	Celery	35	35	3509	3579
8	Grapes_untrained	112	111	11,048	11,271
9	Soil_vinyard_develop	62	61	6080	6203
10	Corn_senesced_green_weeds	32	32	3214	3278
11	Lettuce_romaine_4wk	10	10	1048	1068
12	Lettuce_romaine_5wk	19	19	1889	1927
13	Lettuce_romaine_6wk	9	9	898	916
14	Lettuce_romaine_7wk	10	10	1050	1070
15	Vinyard_untrained	72	71	7125	7268
16	Vinyard_vertical_trellis	18	17	1772	1807
Total		533	527	53,069	54,129

**Table 3:** Sample division results for the PU dataset.

No.	Class	Train	Validation	Test	Total
1	Asphalt	66	65	6500	6631
2	Meadows	186	184	18,279	18,649
3	Gravel	20	20	2059	2099
4	Trees	30	30	3004	3064
5	Painted metal sheets	13	13	1319	1345
6	Bare soil	50	49	4930	5029
7	Bitumen	13	13	1304	1330
8	Self-blocking bricks	36	36	3610	3682
9	Shadows	9	9	929	947
Total		423	419	41,934	42,776

**Table 4:** Experimental results of ablation experiments on three datasets. Please note that “√” and “×” represent whether the module is selected and not selected in the model, respectively.

Datasets	MSIE	SGA	Standard Convolution	OA	AA	Kappa
IP	*√*	×	×	97.67	90.60	97.34
	×	*√*	*√*	81.05	80.16	78.34
	*√*	*√*	×	98.24	94.64	97.99
SA	*√*	×	×	97.16	98.27	97.16
	×	*√*	*√*	84.47	85.51	82.58
	*√*	*√*	×	97.89	98.44	97.65
PU	*√*	×	×	97.82	96.69	97.10
	×	*√*	*√*	95.83	94.30	94.49
	*√*	*√*	×	98.34	96.82	97.80

**Table 5:** Classification accuracy results of different models on the IP dataset. Please note that bold indicates the highest classification accuracy.

No.	DFFN	HSST	SSAN	SSFTTnet	MASSFormer	MSSTT	SGTN
1	**89.48 ± 7.75**	0.00 ± 0.00	67.54 ± 10.16	62.28 ± 14.30	80.70 ± 5.41	30.70 ± 4.47	79.82 ± 8.13
2	97.04 ± 1.39	44.42 ± 19.31	90.93 ± 5.03	93.44 ± 1.85	89.35 ± 2.56	77.09 ± 6.09	**97.95 ± 0.45**
3	96.93 ± 0.54	77.32 ± 7.49	96.03 ± 2.03	95.64 ± 1.40	96.14 ± 1.79	72.66 ± 8.44	**98.02 ± 0.19**
4	**100.00 ± 0.00**	56.48 ± 8.17	97.06 ± 2.82	94.65 ± 1.36	97.06 ± 2.41	73.40 ± 5.94	98.96 ± 1.12
5	97.36 ± 1.56	26.02 ± 13.93	94.56 ± 1.89	96.85 ± 1.73	91.07 ± 5.90	86.57 ± 7.44	**99.57 ± 0.32**
6	96.39 ± 1.17	96.11 ± 1.31	**99.38 ± 0.48**	94.43 ± 0.69	97.58 ± 0.84	95.27 ± 0.84	97.75 ± 0.48
7	41.67 ± 3.40	0.00 ± 0.00	36.11 ± 10.39	**68.06 ± 12.88**	56.94 ± 5.20	19.44 ± 21.87	62.50 ± 18.94
8	100.00 ± 0.00	100.00 ± 0.00	98.80 ± 1.00	99.91 ± 0.12	99.83 ± 0.12	100.00 ± 0.00	**100.00 ± 0.00**
9	74.51 ± 10.00	0.00 ± 0.00	35.30 ± 8.32	52.94 ± 24.01	84.32 ± 10.00	25.49 ± 18.18	**92.16 ± 7.34**
10	95.18 ± 1.20	59.01 ± 20.20	94.12 ± 1.56	92.47 ± 2.72	92.89 ± 1.32	66.50 ± 2.02	**97.84 ± 0.81**
11	97.82 ± 0.94	90.60 ± 2.82	98.51 ± 0.20	95.64 ± 1.51	94.97 ± 0.95	88.35 ± 3.19	**98.57 ± 0.13**
12	95.70 ± 1.42	42.14 ± 30.42	91.20 ± 3.28	93.76 ± 3.00	93.14 ± 1.11	65.21 ± 4.97	**98.68 ± 0.20**
13	99.80 ± 0.28	88.43 ± 5.97	**100.00 ± 0.00**	100.00 ± 0.00	98.80 ± 1.69	99.40 ± 0.49	99.80 ± 0.28
14	98.99 ± 0.25	**99.35 ± 0.41**	97.53 ± 0.44	97.30 ± 0.56	96.23 ± 0.88	96.36 ± 2.17	98.73 ± 0.50
15	97.03 ± 0.84	64.65 ± 4.53	95.97 ± 2.99	91.93 ± 3.81	94.69 ± 1.43	82.91 ± 1.69	**97.88 ± 1.17**
16	**100.00 ± 0.00**	0.00 ± 0.00	60.96 ± 43.51	91.23 ± 5.51	95.61 ± 0.62	83.77 ± 5.08	96.05 ± 1.86
OA	97.22 ± 0.28	72.66 ± 0.43	95.29 ± 1.26	94.87 ± 0.16	94.29 ± 0.49	83.08 ± 0.97	**98.24 ± 0.14**
AA	92.37 ± 1.15	52.78 ± 1.48	84.63 ± 2.67	88.79 ± 3.16	91.21 ± 1.42	72.69 ± 2.03	**94.64 ± 1.32**
Kappa × 100	96.83 ± 0.32	68.44 ± 0.68	94.62 ± 1.44	94.15 ± 0.19	93.49 ± 0.56	80.58 ± 1.16	**97.99 ± 0.16**

**Table 6:** Classification accuracy results of different models on the SA dataset. Please note that bold indicates the highest classification accuracy.

No.	DFFN	HSST	SSAN	SSFTTnet	MASSFormer	MSSTT	SGTN
1	**100.00 ± 0.00**	0.00 ± 0.00	99.36 ± 0.91	99.88 ± 0.09	99.81 ± 0.17	94.45 ± 2.91	99.85 ± 0.22
2	96.18 ± 2.91	99.76 ± 0.28	97.84 ± 2.15	99.48 ± 0.36	95.16 ± 5.58	99.91 ± 0.07	**100.00 ± 0.00**
3	90.66 ± 5.28	31.34 ± 20.12	92.07 ± 2.39	98.04 ± 0.37	97.01 ± 1.13	72.62 ± 5.29	**98.36 ± 1.17**
4	98.44 ± 1.39	77.68 ± 29.71	97.83 ± 1.33	98.32 ± 1.37	**98.98 ± 0.52**	97.10 ± 0.48	98.08 ± 1.34
5	**99.75 ± 0.24**	95.55 ± 3.23	95.79 ± 0.95	99.25 ± 0.76	99.52 ± 0.11	94.93 ± 1.89	99.26 ± 0.94
6	99.99 ± 0.01	99.96 ± 0.04	**99.99 ± 0.01**	99.96 ± 0.04	99.95 ± 0.06	99.90 ± 0.06	99.98 ± 0.01
7	97.00 ± 1.91	**99.50 ± 0.18**	97.66 ± 1.66	97.85 ± 0.29	96.31 ± 1.48	93.13 ± 1.58	99.45 ± 0.18
8	93.04 ± 2.29	95.76 ± 4.91	90.76 ± 0.65	85.81 ± 2.52	88.24 ± 2.44	79.40 ± 5.86	**96.07 ± 1.01**
9	99.79 ± 0.07	99.86 ± 0.13	99.27 ± 0.09	99.51 ± 0.61	99.79 ± 0.15	99.59 ± 0.15	**99.86 ± 0.05**
10	97.46 ± 1.30	96.76 ± 2.34	97.23 ± 1.60	97.24 ± 2.37	95.41 ± 0.11	91.30 ± 0.89	**98.93 ± 0.37**
11	98.28 ± 1.10	0.86 ± 1.22	94.21 ± 1.52	95.74 ± 1.61	93.13 ± 1.64	81.65 ± 2.60	**99.77 ± 0.18**
12	98.13 ± 1.72	92.50 ± 8.41	**99.91 ± 0.12**	99.17 ± 0.99	98.96 ± 0.87	99.65 ± 0.32	99.42 ± 0.27
13	99.15 ± 1.21	71.94 ± 13.33	98.66 ± 0.63	**99.59 ± 0.43**	99.11 ± 0.64	98.59 ± 0.14	98.33 ± 0.66
14	99.24 ± 0.20	78.60 ± 10.28	96.95 ± 0.56	99.24 ± 0.21	98.73 ± 0.59	97.33 ± 0.21	**99.59 ± 0.58**
15	78.63 ± 12.52	5.53 ± 7.53	92.58 ± 7.35	85.30 ± 8.34	87.91 ± 2.97	82.77 ± 12.72	**94.42 ± 2.30**
16	**97.20 ± 3.08**	71.24 ± 14.51	97.20 ± 3.30	94.73 ± 1.09	95.96 ± 0.90	87.10 ± 0.89	93.68 ± 3.18
OA	94.42 ± 1.56	75.07 ± 0.61	95.65 ± 1.00	94.20 ± 0.69	94.53 ± 0.39	89.94 ± 0.90	**97.89 ± 0.22**
AA	96.43 ± 1.00	69.80 ± 1.66	96.71 ± 0.52	96.82 ± 0.52	96.50 ± 0.34	91.84 ± 0.91	**98.44 ± 0.31**
Kappa × 100	93.78 ± 1.74	71.85 ± 0.73	95.16 ± 1.11	93.55 ± 0.77	93.91 ± 0.42	88.81 ± 1.01	**97.65 ± 0.25**

**Table 7:** Classification accuracy results of different models on the PU dataset. Please note that bold indicates the highest classification accuracy.

No.	DFFN	HSST	SSAN	SSFTTnet	MASSFormer	MSSTT	SGTN
1	97.60 ± 1.94	82.06 ± 5.90	92.75 ± 1.41	95.43 ± 0.42	93.40 ± 2.96	**99.47 ± 0.18**	98.50 ± 0.16
2	98.31 ± 1.48	96.93 ± 0.47	97.42 ± 1.24	97.66 ± 0.83	97.14 ± 0.75	98.25 ± 0.48	**99.70 ± 0.39**
3	93.88 ± 1.93	20.24 ± 13.61	67.87 ± 5.08	85.25 ± 3.35	67.54 ± 6.67	83.42 ± 1.95	**95.81 ± 0.48**
4	93.02 ± 1.95	**97.39 ± 0.26**	88.44 ± 0.79	93.11 ± 0.72	93.76 ± 1.36	87.97 ± 1.16	92.14 ± 0.15
5	99.87 ± 0.18	99.52 ± 0.22	**99.97 ± 0.04**	99.60 ± 0.34	99.92 ± 0.06	99.72 ± 0.03	99.85 ± 0.12
6	91.13 ± 2.33	29.27 ± 13.33	92.10 ± 2.36	87.46 ± 5.03	77.01 ± 4.52	88.85 ± 1.11	**98.15 ± 1.12**
7	94.30 ± 5.20	71.01 ± 19.91	78.09 ± 3.86	80.06 ± 6.02	75.51 ± 8.98	92.87 ± 0.67	**98.19 ± 0.80**
8	96.98 ± 2.18	98.96 ± 0.55	93.24 ± 1.09	93.53 ± 3.99	94.58 ± 3.68	98.62 ± 0.24	**99.91 ± 0.01**
9	88.34 ± 1.72	82.96 ± 6.99	72.73 ± 9.80	91.28 ± 0.97	**96.52 ± 1.91**	86.40 ± 1.15	89.13 ± 4.08
OA	96.35 ± 0.83	82.08 ± 0.60	92.55 ± 1.07	94.20 ± 0.71	91.68 ± 0.57	95.52 ± 0.16	**98.34 ± 0.17**
AA	94.83 ± 0.35	75.37 ± 3.13	86.95 ± 1.28	91.49 ± 1.08	88.38 ± 0.20	92.84 ± 0.18	**96.82 ± 0.38**
Kappa × 100	95.16 ± 1.08	75.57 ± 1.03	90.12 ± 1.41	92.29 ± 0.96	88.89 ± 0.73	94.03 ± 0.21	**97.80 ± 0.22**

**Table 8:** Analysis of parameters and testing time for different models on three datasets.

Model	IP		SA		PU	
	Parameters (M)	Testing Time (s)	Parameters (M)	Testing Time (s)	Parameters (M)	Testing Time (s)
DFFN	0.49	1.73	0.49	10.63	0.49	4.37
HSST	0.26	1.95	0.26	15.67	0.26	12.4
SSAN	2.97	1.27	3.88	7.44	3.88	6.01
SSFTTnet	0.03	0.91	0.03	5.11	0.03	4.11
MASSFormer	0.19	0.95	0.19	5.76	0.19	4.65
MSSTT	0.55	3.37	0.53	25.17	0.16	9.53
SGTN	0.22	1.52	0.22	5.93	0.22	4.89

## Data Availability

Datasets used within this study are available upon request.
